# Identification and Alternative Splicing Profile of the *Raffinose synthase* Gene in Grass Species

**DOI:** 10.3390/ijms241311120

**Published:** 2023-07-05

**Authors:** Junhao Xu, Xiangkai You, Yanan Leng, Youyue Li, Zeyu Lu, Yinan Huang, Moxian Chen, Jianhua Zhang, Tao Song, Tieyuan Liu

**Affiliations:** 1 College of Grassland Agriculture, Northwest A&F University, Yangling 712100, China; 2 Co-Innovation Center for Sustainable Forestry in Southern China, College of Biology and the Environment, Nanjing Forestry University, Nanjing 210000, China; 3 Department of Biology, Hong Kong Baptist University, Kowloon, Hong Kong; 4 State Key Laboratory of Agrobiotechnology, The Chinese University of Hong Kong, Shatin, Hong Kong

**Keywords:** raffinose synthase, grass species, gene family, alternative splicing, drought and salt stress

## Abstract

*Raffinose synthase* (*Rafs*) is an important enzyme in the synthesis pathway of raffinose from sucrose and galactinol in higher plants and is involved in the regulation of seed development and plant responses to abiotic stresses. In this study, we analyzed the *Rafs* families and profiled their alternative splicing patterns at the genome-wide scale from 10 grass species representing crops and grasses. A total of 73 *Rafs* genes were identified from grass species such as rice, maize, foxtail millet, and switchgrass. These *Rafs* genes were assigned to six groups based the phylogenetic analysis. We compared the gene structures, protein domains, and expression patterns of *Rafs* genes, and also unraveled the alternative transcripts of them. In addition, different conserved sequences were observed at these putative splice sites among grass species. The subcellular localization of *PvRafs5* suggested that the *Rafs* gene was expressed in the cytoplasm or cell membrane. Our findings provide comprehensive knowledge of the *Rafs* families in terms of genes and proteins, which will facilitate further functional characterization in grass species in response to abiotic stress.

## 1. Introduction

Raffinose family oligosaccharides (RFOs) are a class of oligosaccharides prevalent in higher plants [1]. Raffinose, a trisaccharide consisting of fructose, glucose, and galactose, is unique to plants and is second only to sucrose in terms of its content in plants [2]. Evidence is mounting that raffinose not only accumulates in seeds, improving seed desiccation tolerance and promoting seed development, but is also largely synthesized in leaves and roots as an important protective mechanism against abiotic stress in higher plants [3,4,5,6]. Metabolic studies, for example, have demonstrated that raffinose accumulated in large amounts in the roots of *Panicum virgatum* under drought stress [7]. The *ZmRAFS* gene is overexpressed in *Zea mays* during drought to increase raffinose content to control leaf water loss and improve drought tolerance [8]. When *Coffea arabica* L. is subjected to abiotic stress, a large amount of raffinose accumulates to resist the stress [9]. Furthermore, theoretical studies have revealed that the accumulation of raffinose in plants is increased during stress conditions to offset the accumulation of reactive oxygen species (ROS) [10,11,12], maintain the normal progress of photosynthesis [13], protect the stability of cell membrane phospholipids [14], and stabilize cell turgor pressure [2,15,16]. Additionally, raffinose can also be transported to chloroplasts to protect thylakoids and maintain the stability of PSII [17]. Apart from the beneficial effects on plant growth and stress tolerance, raffinose can also promote the growth of beneficial bacteria, such as *Bifidobacteria* and *Lactobacilli* [18,19,20]. Thus, raffinose has been considered to have multifunctional benefits to plants and microbiomes.

The synthesis of raffinose requires two enzymatic reactions. First, galactinol synthase (Gols, EC 2.4.1.123) uses UDP-galactose and inositol as substrates to synthesize inositol galactoside [2]. Then, raffinose synthase (*Rafs*, EC 2.4.1.82) uses inositol galactoside and sucrose as substrates to synthesize raffinose [21]. Thus, genetic technologies, for example, overexpression or knockout of these two genes, have been the main method to determine the function of raffinose. Multiple *Gols* isoforms have been characterized from *Arabidopsis thaliana* or other legume plants, and these *Gols* genes were proven to improve plant abiotic stress by regulating raffinose accumulation [22,23]. *Rafs* is a key enzyme in controlling the last step in raffinose synthesis. The *Rafs* gene was first reported to be cloned from *Pisum sativum* [24]. Later, *Rafs* coding sequences were cloned in *Cucumis statirus* [25], *Oryza sativa* [26], and *Arabidopsis thaliana* [21]. Despite the identification and functional study of *Rafs* in Arabidopsis and main crops [27], the *Rafs* families in grass species and how they respond to abiotic stress such as drought stress remain scant.

Alternative splicing (AS) expands the complexity of proteins and phenotypic traits by generating multiple mRNA transcripts from one gene, which has important functions in different developmental processes in plants. For example, in Arabidopsis, the abundance of the *MAF2* var2 transcript decreased after cold treatment, whereas the abundance of the *MAF2* var1 transcript as a repressor of flowering increased [28]. *MaMYB16L* is a transcription repressor in *Musa nana* Lour. fruit and produces two transcripts, the full-length isoform *MaMYB16L* and the truncated spliced form *MaMYB16S*. *MaMYB16L* negatively regulates banana fruit ripening by directly inhibiting the transcription of starch degradation and many genes. *MaMYB16S* can form a dimer with *MaMYB16L* to reduce the activity of *MaMYB16L* [29]. At the protein level, AS is able to determine subcellular localization, biological, or biochemical functions of proteins. The YUCCA4, which is required for auxin biosynthesis, could produce two alternative splice variants. One protein isoform was localized in the cytosolic side of the endoplasmic reticulum with a transmembrane domain, whereas the other isoform was anchored to the cytosol [30]. The wheat *TaNAK1* gene has key functions in encoding the NB-ARC domain and protein kinase domain protein, which is also subject to AS and produces two splicing variants. Subcellular localization suggests that *TaNAK1.1* was mainly localized in the cytoplasm, while *TaNAK1.2* was localized in the nucleus and cytoplasm. Despite both the two isoforms displaying protein kinase activity in vitro, the opposite function in regulating flowering time and plant architecture were observed between the two splicing variants [31]. However, the AS of *Rafs* families in generating transcript diversity has received relatively little attention.

In this study, we identified the *Rafs* gene family from 10 grass plants for evolution, gene structure, protein domain/motif analysis, AS profile analysis, and expression pattern analysis to reveal the characteristics of these gene families in grasses, provide references for further study of *Rafs* function, and discuss their roles in stress resistance.

## 2. Result

### 2.1. Identification and Phylogenetic Analysis of Rafs Families in 10 Grass Species

To identify possible *Rafs* genes throughout the grass species, we used the protein sequence *Rafs* (EC 2.4.1.82) to perform BLAST searches in Phytozome v12.0. All sequences with the annotation of *Rafs* were selected and subjected to the online SMART tool for protein domain analysis, and sequences lacking the *Rafs* domain were eliminated. Subsequently, 73 *Rafs* genes were identified from 10 grass species (Table S1), including 5 *Brachypodium distachyon* (purple false brome), 5 *Brachypodium stacei*, 6 *Oropetium thomaeum*, 6 *Oryza sativa* (rice), 7 *Panicum hallii* (hall’s panicgrass), 16 *Panicum virgatum* (switchgrass), 7 *Setaria italica* (foxtail millet), 7 *Setaria viridis* (green foxtail), 7 *Sorghum bicolor* (sorghum), and 7 *Zea mays Ensembl* (maize). Each gene sequence of the species is listed in order according to their scores in Phytozome v12.0. Most of these genes are located on different chromosomes, and only a small part is located on the same chromosome (Table S2). Interestingly, each species has genes located on the same chromosome except the genes of *Brachypodium stacei* and rice. The length of identified genes ranged from 1674 bp to 19,726 bp, which revealed that a fairly short sequence can play an important role. The subcellular localization patterns of all *Rafs* were predicted by the online CELLO. Most members of each species were predicted to be chloroplast and cytoplasmic localized, and other proteins were predicted to be periplasmic, consistent with their roles as photosynthetic products and their functions.

**Figure 1:**
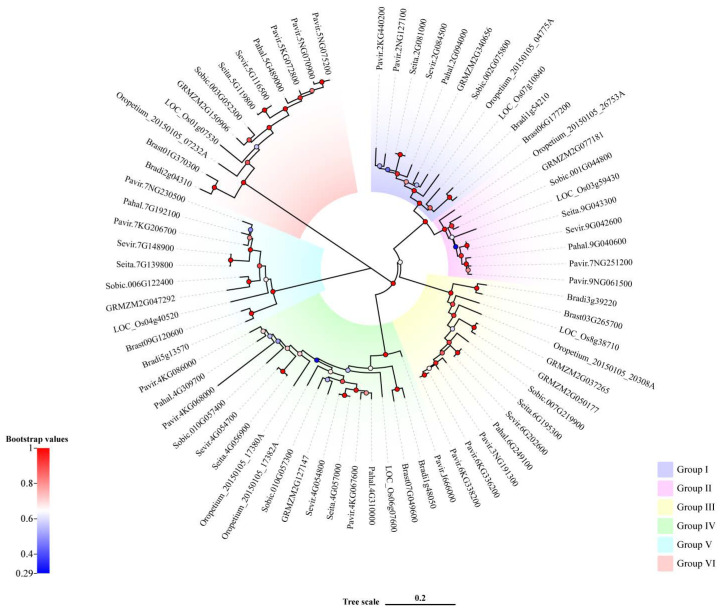
Phylogenetic analysis of the *Rafs* genes in plants. Bootstrap values are presented as color gradients at the branches. Different groups are marked with different colors.

To analyze the evolutionary relationship among members of *Rafs* family, we performed multiple sequence alignment on 73 sequences, and a phylogenetic tree of 73 *Rafs* from 10 species was constructed using Bayesian methods (Figure 1). According to the results, they were divided into 6 clades. The constructed phylogenetic tree was linked to overall high bootstrap values represented by color gradients. Normally, paralogous genes from the same species, such as the three *Rafs* from switchgrass, were grouped together, indicating very limited divergence between them. In general, the high homology of *Rafs* gene sequences indicated that the differentiation of them among different species is relatively conservative. The similarity of protein sequences indicated that *Rafs* gene of these 10 species have the same biological function. In conclusion, the *Rafs* gene is highly conserved in the course of species evolution. The physicochemical characteristics of *Rafs* were analyzed by the ProtParam online analysis tool (Table S3). The molecular weights of *Rafs* in 10 species ranged from 44.06 kDa to 100.38 kDa, which were due to the length of their sequences. The theoretical isoelectric point (pI) is between 5.30 and 9.68; however, the pI of two sequences of *Oropetium thomaeum* cannot be computed because they contain several consecutive undefined amino acid. The proportion of amino acid residues with a negative charge is larger than that with a positive charge in more than 97% of sequences. Except for *Bradi3g39220* of purple false brome, all the other proteins were unstable proteins with an instability index of 65.24~88.71. The grand average of hydropathicity (GRAVY) of most proteins ranged from −0.368 to 0.043, which belongs to amphoteric proteins. The secondary structure of *Rafs* was predicted online by SOPMA. The results showed that the secondary structure consisted mainly of alpha helices and random coils, which were 23.9~36.05% and 39.54~47.35%, respectively. The proportion of extended strand was less, and the Beta turn was the lowest, which was 5.21~9.5%.

### 2.2. Protein Domain and Motif Analysis of Rafs Families in 10 Grass Species

**Figure 2:**
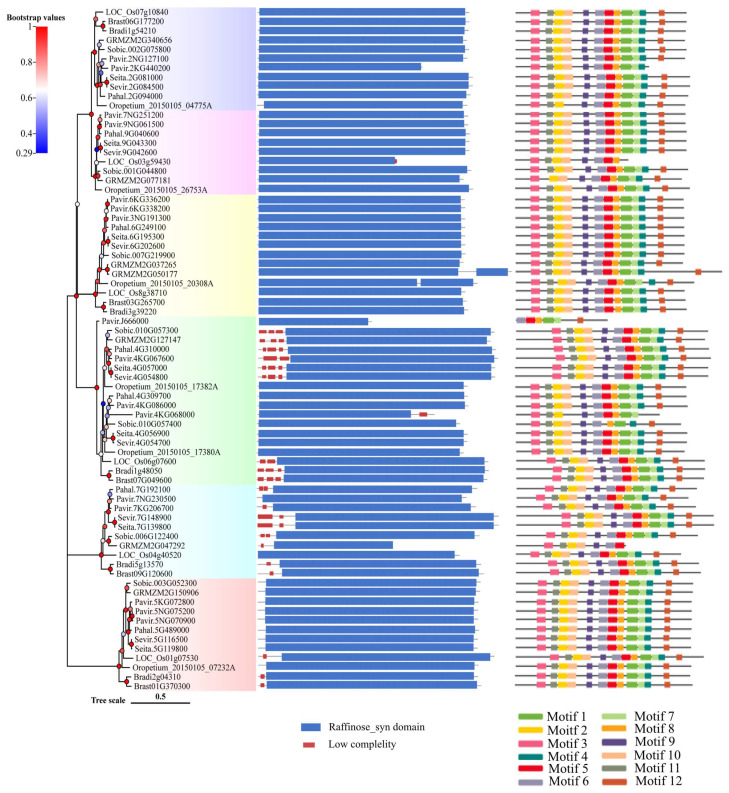
Protein domain and motif analysis of the *Rafs* family in 10 grass species. The phylogenetic relationship is listed on the left panel. Protein domain predicted by the SMART online tool are listed in the middle panel. Conserved motifs analyzed by the MEME online tool are listed in the right panel.

All *Rafs* have a characteristic Raffinose_syn domain, and some members have low-complexity regions at their N- or C-terminal regions according to the results from the online tool SMART (Figure 2, middle panel). Unexpectedly, two Raffinose_syn domains were detected but appeared only two times in *GRMZM2G050177* of maize and *Oropetium_20150105_20308A* of *Oropetium thomaeum*. *Rafs* from all species range from 410 to 922 amino acids, and most of them are approximately 700 amino acids (Table S2). The size of the Raffinose_syn domain is not strictly within a certain range but is different in length. The longest is *Oropetium_20150105_26753A* of *Oropetium thomaeum*, which has 758 amino acids. The wide range of Raffinose_syn domain lengths is also the main reason for the different lengths of *Rafs* sequences in different species. Some *Rafs* proteins have a larger size due to their significantly larger N-terminus or C-terminus.

**Figure 3:**
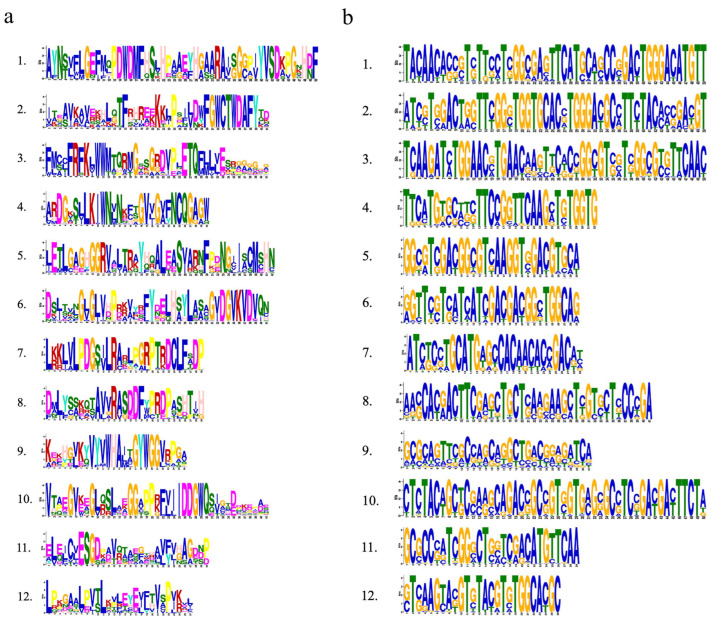
Weblogo plots of conserved motifs in the plant *Rafs* family. (**a**) Conserved motifs in *Rafs* proteins. (**b**) Conserved motifs in the CDS of the *Rafs* genes.

The conserved motif in the *Rafs* protein was analyzed using the online tool Multiple Em for Motif Elicitation (MEME) (Figure 2, right panel and Figure 3a). Twelve conserved motifs are represented by different colored boxes that are able to cover most of the protein. Proteins from all species normally have 12 conserved motifs. Although the number of motifs in different sequences is different, they are consistent in order. In larger scale proteins, such as *GRMZM2G050177* of maize, significantly larger gaps between conserved motifs were observed, indicating the acquisition of novel sequences. The regions roughly corresponding to the Raffinose_syn domain were split into 12 adjacent motifs: motif 1 to motif 12, except *Pavir.2KG440200*, *Oropetium_20150105_04775A* of group 1, *LOC_Os03g59430*, *GRMZM2G077181* of group 2, and *Pavir.J666000*, *Pavir.4KG068000*, *Sobic.010G057400* of group 4, and *GRMZM2G047292* of group 5. Motif three and motif eleven are located at the N-terminal end and are found in most *Rafs* proteins throughout grass species except *GRMZM2G077181* and *Pavir.J666000*. In the C-terminus, there are three motifs (motif four, motif seven, and motif twelve) that exist in most grass species except *LOC_Os03g59430*, *Pavir.J666000* and *GRMZM2G047292*. Intriguingly, all motifs were found in *Rafs* proteins of purple false brome, *Brachypodium stacei*, hall’s panicgrass, foxtail millet, and green foxtail, while proteins of switchgrass, *Oropetium thomaeum*, rice, maize, and sorghum lacked some motifs, suggesting that the variation at this region likely contributed to the divergence between *Rafs* genes in different grass species plants.

### 2.3. Gene Structure and Conserved Motif Analysis of Rafs Families in 10 Grass Species

**Figure 4:**
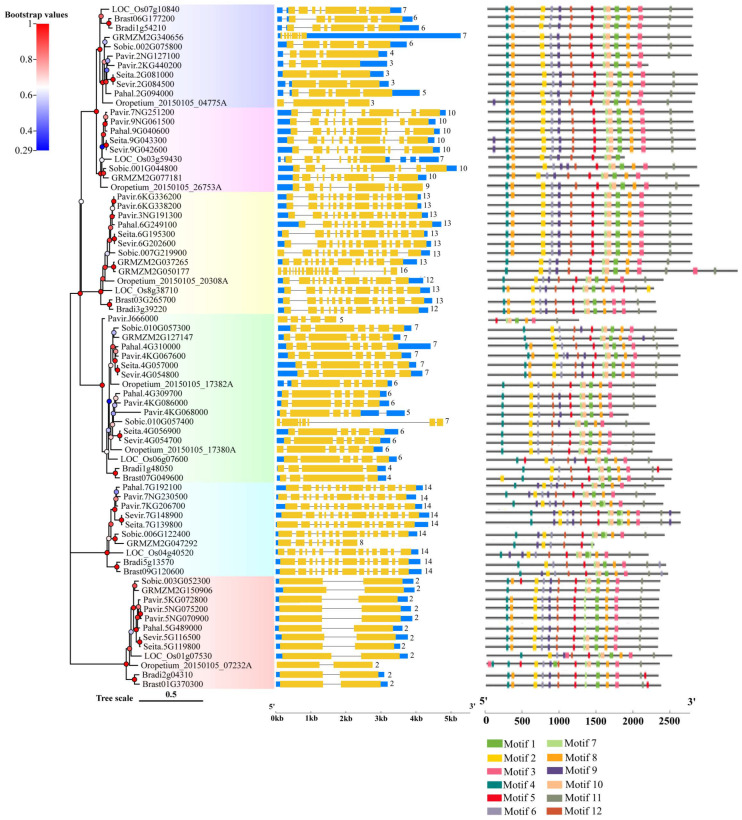
Gene structures and motif analysis in the coding region of *Rafs* genes in 10 grass species. The phylogenetic relationship is listed on the left panel. Exon–intron structures are displayed in the middle panel. Yellow bars indicate coding regions, blue bars indicate untranslated regions, and black lines indicate introns. The number of exons in the coding region is shown at the end of each transcript. Conserved motifs in the coding region, represented by different colored boxes, and placed on the right panel. (The genetic model of *GRMZM2G340656* is scaled down four times, and the genetic models of *Sobic.010G057400* and *GRMZM2G050177* are scaled down two times).

Figure 4 shows the exon–intron structure of each *Rafs* gene transcript with coding sequence. In general, various genome structures were observed with the number of total exons varying from 2 to 16. Each group branch had a similar total number of exons. In the second to sixth groups, the number of exons of most plants remained stable, approximately 10, 13, 6 or 7, 14 and 2, respectively. In contrast, the exons that appeared in group one branch were very different. The *Rafs* genes that have exactly 10, 13, 6 or 7, 14 and 2 exons in their CDS account for approximately 79% of the whole number (Figure 4 and Table S2), including *Rafs* genes from the representative species maize and switchgrass. Thus, the X exon-(X-1) intron organization appears to represent the conserved gene structure of most *Rafs* genes in all branches of the grass species lineage groups. Notably, some genes contain introns in their 5′ or 3′ untranslated (UTR) regions, suggesting potential variations in the control of their transcriptional or translational efficiency. An extreme example is maize *GRMZM2G340656*, which has a long 3′ UTR, although its CDS still has seven exons.

The CDS of the *Rafs* gene was also analyzed by the MEME program (Figure 4, right panel and Figure 3b). As shown in Figure 2, the conserved patterns predicted in the CDS are similar to those in the proteins. In general, the number and pattern of motifs remained similar between most grass species, while the *Rafs* genes of *LOC_Os3g59430* of group 2, *Pavir.J666000* of group 4, and *GRMZM2G047292* of group 5 have significantly fewer motifs. The lower number of exons may be caused by exon fusions without loss of conserved motifs, as in *Oropetium_20150105_26753A* (nine exons) of *Oropetium thomaeum*. Furthermore, genes from group 6, such as *Pavir.5NG070900*, *Pavir.5NG075200*, *Pahal.5G489000*, etc., have a relatively normal motif in their CDS but possess only one intron, probably caused by reverse transcriptase-mediated gene duplication. On the other hand, proteins with larger volumes tend to have additional exons in their CDS that do not occur in most other genes. One example is that the two Raffinose_syn structural domains containing *GRMZM2G050177* originally had 16 exons; therefore, the extra exons may help explain the acquisition of the new protein structural domain.

Although the CDS size of *Rafs* genes is relatively stable, the length of introns is highly variable, resulting in a large range of genome sizes for this family (Table S2). Although the *Rafs* genes of maize have the shortest genome length of approximately 2300 bp, the genome lengths of some genes are enormous, exceeding 19,000 bp, such as *GRMZM2G340656*. Because of the large variation in intron length and differences in UTR regions, gene structure sometimes does not appear to correlate well with phylogenetic relationships inferred from protein sequences. For example, different patterns were observed in closely grouped genes such as *Sobic.010G057300* and *Sobic.010G057400* of sorghum.

### 2.4. Cis-Regulatory Element Identification in the Promoter Region of Rafs Families

**Figure 5:**
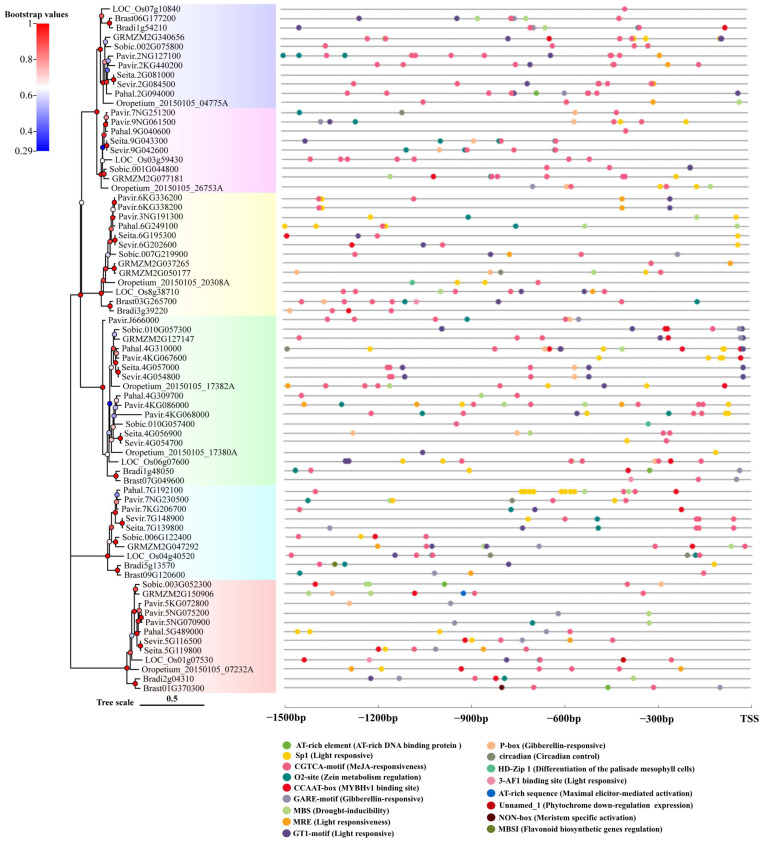
Analysis of putative cis-regulatory elements in the promoter region of the *Rafs* gene in 10 grass species. Seventeen tissue-related and hormone-related cis-regulatory elements are presented by colored circles. TSS: transcription starting site.

To analyze the transcriptional regulation of plant *Rafs* genes, 1500 bp genomic sequences upstream of the *Rafs* transcription start site were analyzed using the online software PlantCare. As a result, various potential cis-regulatory elements were identified and are shown in Figure 5. The number of cis-elements varied greatly among the different *Rafs* genes. Among all the genes, *Pahal.7G192100* of hall’s panicgrass has the maximum number of 15 motifs, while *Seita.2G081000* of foxtail millet contain no motif analyzed by the program noted above (Figure 5).

In total, four light responsive (i.e., 3-AF1 binding site, GT1-motif, Sp1, and MRE) cis-regulating elements were detected from all promoters. They are common in that more than 75% of the sequences have at least one of them. GT1-motif and Sp1 occur most frequently, and the vast majority of promoters contain these motifs. MRE is relatively rare, and the 3-AF1 binding site is only present in *Brast03G265700*, *Brast07G049600* and *LOC_Os01g07530*. Aunamed cis-regulating elements related to phytochrome downregulation expression were found in *LOC_Os01g07530*. A total of three hormone-specific motifs were identified, including methyl jasmonate (MeJA), and gibberellin-specific motifs (i.e., GARE-motif, P-box, CGTCA-motif). Motifs associated with MeJA and gibberellin are the most prevalent and can be found in more than 94% of promoters. Some promoters of *Rafs* genes have only one hormone-specific motif, such as *Bradi5g13570*, which has only MeJA-responsive (i.e., CGTCA-motif) motif, while some other promoters contain motifs related to various hormones. For example, patterns related to all hormones can be found in the promoter of *Pavir.9NG061500*. Other motifs (O2-site, CCAAT-box, and MBS) are also common, as more than 68% of the sequences have at least one, and MBS is related to drought inducibility. Some cis-regulatory elements were found only in one or two sequences, but they played an important role in plants. MBSI was found in *Bradi5g13570*, which was related to the regulation of flavonoid biosynthesis genes. NON-box was found in *Brast01G370300*, which was related to meristem-specific activation.

Although most motifs can be detected in *Rafs* genes across different species of grass, some are enriched in a particular plant lineage. For example, similar motif patterns were observed in promoters between some closely related homologous genes, such as *Seita.4G057000* and *Sevir.4G054800*, *Seita.9G043300* and *Sevir.9G042600*, and *Pavir.6KG336200* and *Pavir.6KG338200*, which provide evidence that the functions of these genes are closely related. Interestingly, similar *Rafs* genes from the same species may show different numbers and patterns of motifs, suggesting functional diversity among duplicated genes. For example, the promoter of *GRMZM2G050177* has six regulatory motifs, while *GRMZM2G037256* has only two, implying that this gene copy may not be as actively transcribed as the other gene.

### 2.5. Protein Conservation Analysis and Interaction Networks of Rafs

**Figure 6:**
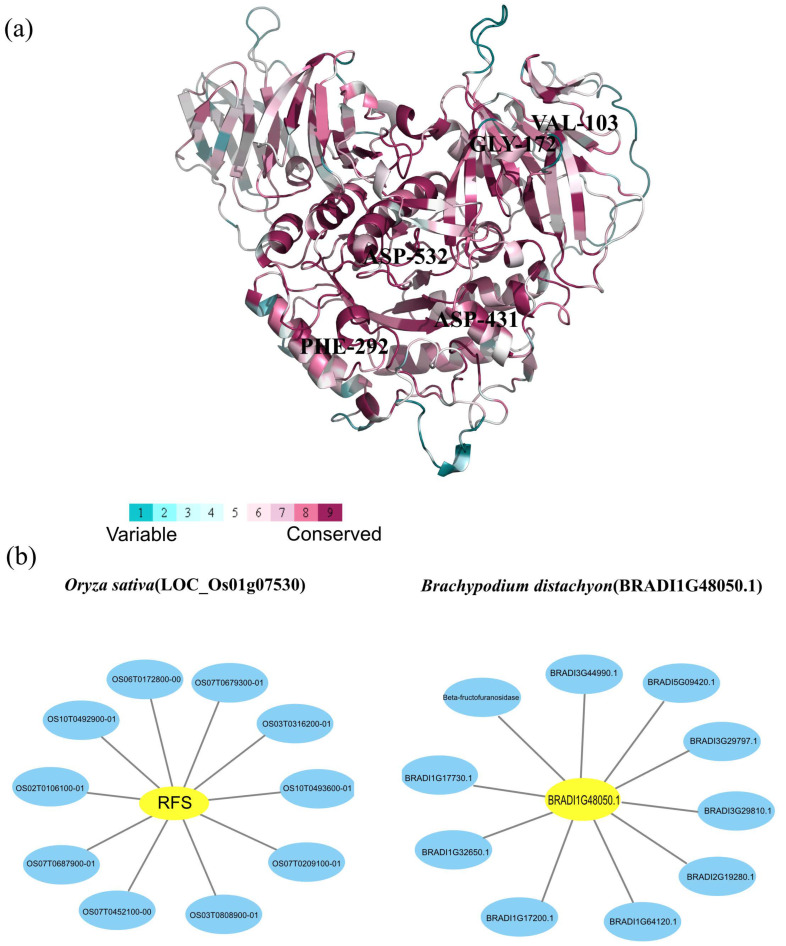
Analysis of amino acid conservation and protein interactions. (**a**) Evolutionary conservation analysis of amino acids in *Rafs*. The ribbon representation is colored according to the ConSurf Grade (1-blue to 9-purple) using all identified protein sequences. (**b**) Representative protein–protein interaction networks of Oryza sativa and Brachypodium distachyon. Interaction networks of *Rafs* (highlighted in yellow) of (**left**) *Oryza sativa* (*LOC_Os01g07530*) and (**right**) *Brachypodium distachyon* (*BRADI1G48050*.1) were constructed by Cytoscape 3.9.1 software based on experiments and databases from the STRING database. Interactors are highlighted in blue.

Since the AlphaFold structure of the rice *Rafs* (AF-Q5VQG4-F1) is publicly available, evolutionary conservation analysis was based on this structure and identified protein sequences (Figure 6a). An amount of 246 residues were conserved at ConSurf Grade 9 in plants, suggesting the high conservation of *Rafs* in plants. The residues corresponding to Val103 of switchgrass were placed by Ala in *Pavir.7NG230500* and *Pavir.7KG206700*, and Gly172 was also changed to Ala in these genes. Phe292 was mutated to Gly in *Pavir.4GK068000*. Asp431 was replaced by Asn in *Pavir.6KG336200.1* and *Pavir.6KG338200.1*, and Asp532 was replaced by Glu in *Pavir.7KG206700*. Mutations in these genes may mean changes in the function of *Rafs* in switchgrass.

The Systematic analysis of interaction networks plays an important role in understanding the functional mechanism of proteins in biological systems. To investigate the functional relationships between proteins, protein–protein interaction networks of *Rafs* were constructed using the online tool STRING. In this work, we selected two representative protein sequences of *Rafs* from rice and purple false brome to generate interaction networks based on experiments and databases (Figure 6b). First, the 10 predicted interacting proteins of rice *Rafs* protein were extracted, including Os07g0679300 protein (*OS07T0679300-01*), OS10T0493600 (*OS10T0493600-01*), Os03g0316200 protein (*OS03T0316200-01*), Os07g0687900 protein (*OS07T0687900-01*), Os06g0172800 protein (*OS06T0172800-00*), OS10T0492900 (*OS10T0492900-01*), Os02g0106100 protein (*OS02T0106100-01*), OS07T0452100 (*OS07T0452100-00*), OS03T0808900 (*OS03T0808900-01*) and Os07g0209100 protein (*OS07T0209100-01*). The Os07g0679300 protein may be a putative alpha-galactosidase. In addition, it may be involved in cDNA cloning. OS10T0493600 has a role as an alpha-galactosidase and is involved in hydrolyzing raffinose. The Os03g0316200 protein has a role as a hexosyltransferase protein or galactinol synthase 3. The Os07g0687900 protein is involved in water stress induction and cDNA cloning, and the Os06g0172800 protein is involved in unknown biological processes. OS10T0492900 and OS07T0452100 are putative expression of alpha-galactosidase. The Os02g0106100 protein is a vacuolar acid invertase and belongs to the glycosyl hydrolase 32 family. Moreover, OS03T0808900 is related to *Rafs* or seed imbibition protein Sip1-containing protein expression, and Os07g0209100 protein is a putative Sip1 protein. Similarly, 10 predicted interacting proteins of purple false brome *Rafs* protein were extracted, including BRADI1G17200 (*BRADI1G17200.1*), BRADI1G32650 (*BRADI1G32650.1*), BRADI1G64120 (*BRADI1G64120.1*), BRADI2G19280 (*BRADI2G19280.1*), Beta-fructofuranosidase (*BRADI1G09500.1*), BRADI1G17730 (*BRADI1G17730.1*), BRADI3G29797 (*BRADI3G29797.1*), BRADI3G29810 (*BRADI3G29810.1*), BRADI3G44990 (*BRADI3G44990.1*), and BRADI5G09420 (*BRADI5G09420.1*). BRADI1G17200 and BRADI1G64120 are both proteins that are inositol 3-alpha-galactosyltransferases and belong to the glycosyltransferase 8 family. BRADI1G32650 and BRADI2G19280 are probable alpha-glucosidase os06g0675700. Beta-fructofuranosidase is involved in insoluble isoenzyme 3 and belongs to the glycosyl hydrolase 32 family. BRADI3G44990 and BRADI5G09420 are related to beta-fructofuranosidase and are involved in insoluble isoenzyme 1 and insoluble isoenzyme 2, respectively. BRADI1G17730, BRADI3G29797 and BRADI3G29810 are involved in unknown biological processes. Generally, the major function of *Rafs* proteins of rice as well as purple false brome and their interacting proteins are related to the synthesis of raffinose and the metabolism of carbohydrates.

### 2.6. AS Profile of Rafs Families in 10 Grass Species

**Figure 7:**
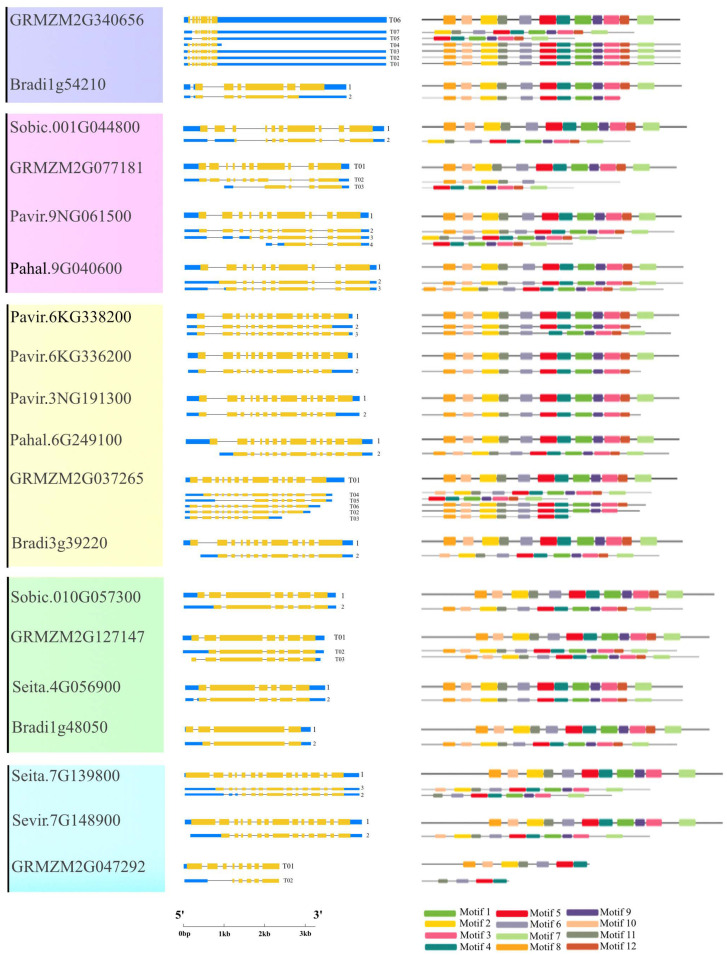
Alternative splicing profile of *Rafs* genes in 10 grass species. Alternative transcripts detected from 19 *Rafs* genes are listed in the middle panel. The protein encoded by each primary transcript is listed to the right of the corresponding transcript with conserved protein motifs illustrated (the same as in Figure 2). Protein isoforms that exhibit different motif patterns deduced by the other alternative transcripts are put below, and identical protein isoforms are only shown once. (* The genetic model of *GRMZM2G340656* is scaled down four times).

We noted that many *Rafs* genes have been annotated with alternative transcripts. To analyze the AS profiles of *Rafs* genes in 10 grasses, we extracted all available alternative transcripts of *Rafs* genes from the Phytozome v12.0 database. As shown in Figure 7, 19 primary transcripts and 35 alternative transcripts were detected in the 19 genes from group 1 to group 4 and group 5 but not in group 6. Of the 19 genes mentioned above, one alternative transcript was detected for each gene in 11 genes, and two alternative transcripts were detected for each gene in 5 genes. Three to six alternative transcripts were detected for each of the three genes. On average, there were one to two alternative transcripts per gene, while *GRMZM2G340656* in maize had the highest number with six alternative transcripts. The primary transcript for each gene as specified by the Phytozome database or other sources is placed at the top, and other so-called alternative transcripts are listed below. The transcripts with the longest CDS, which were used to construct the phylogenetic tree, were identical to the major transcripts listed here. The conserved protein motifs corresponding to the major isoforms as well as different protein isoforms deduced from other alternative transcripts are shown on the right of Figure 7. Protein isoforms with different motif patterns deduced from other alternative transcripts are listed below. Major transcripts typically encode full-length proteins carrying the most conserved protein motifs, whereas alternative transcripts are typically shorter in length and lack certain motifs.

**Figure 8:**
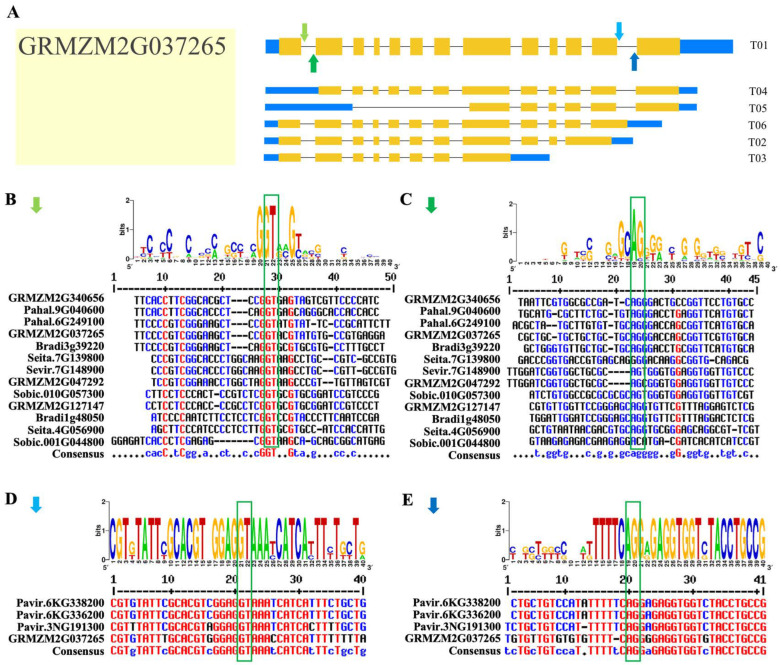
Conserved alternative splice site sequence analysis of plant *Rafs* genes. (**A**) Four splice sites found among the plant *Rafs* family in representative *Zea mays* (*GRMZM2G037265*) were marked with light green arrows (**B**), dark green arrows (**C**), light blue arrows (**D**), and dark blue arrows (**E**). The label for the *y*-axis is “bits”. Red, blue, and black of multiple sequence alignments represent high consensus (default value: 90%), low consensus (default value: 50–90%) and neutral, respectively.

We identified four conserved splice sites at the exon–intron boundaries (i.e., 20 bp on each side) of the *Rafs* gene using Weblogo and multiple alignment analysis. The four conserved splicing sites were marked in Figure 8A with solid arrows of four different colors by *GRMZM2G037265*, a *Rafs* gene in a representative plant of maize. Figure 8B and Figure 8D show the 5′ splice site (5′SS), and Figure 8C and Figure 8E show the 3′ splice site (3′SS). Thirteen *Rafs* genes shared similar 5′ss and 3′ss, while the other four *Rafs* genes also had similar 5′ss and 3′ss, and their splicing sites were roughly at both ends of the *GRMZM2G037265* sequence. In addition, we obtained the degree of identity of their conserved splice site sequences through multiple sequence alignment. The splice site sequences in panels B, D, and E have high identity, while the conserved splice site sequences in panel C have low identity.

### 2.7. Expression Profile Analysis of Rafs Families in Different Tissues and under Various Stress Conditions and Subcellular Localization of the Rafs Protein

**Figure 9:**
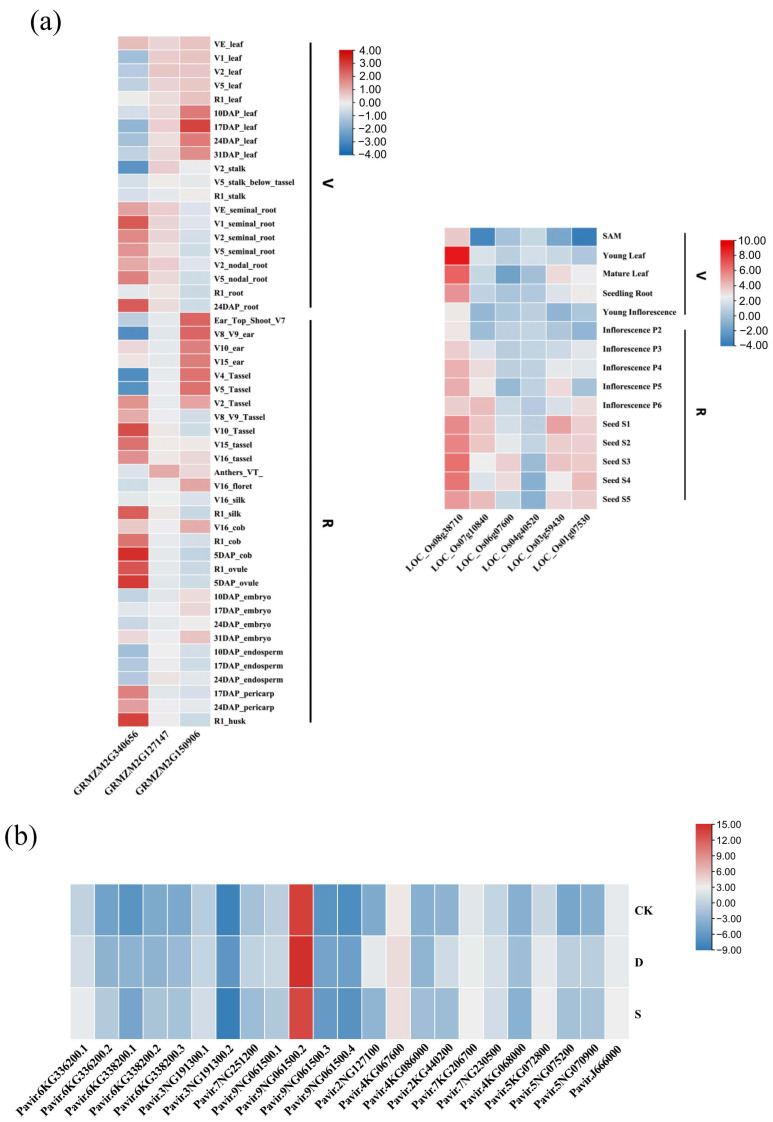
Expression profile of *Raf* genes in representative species. (**a**) Spatial and temporal expression of *Rafs* genes from *Zea mays* (maize) and *Oryza sativa* (rice). V: vegetative organs; R: reproductive organs. Red indicates high levels of transcript abundance, and blue indicates low transcript abundance. The color scales are shown on the right. (**b**) Expression profiles of switchgrass *Rafs* genes under drought and salt stress (the expression of *Pavir.6KG336200.1* was taken as a 0 value).

Tissue-specific expression patterns of several representative *Rafs* genes from the model plants maize and rice were summarized using microarray data from the eFP browser (Figure 9a). For *Rafs* in maize, there is a difference between high expression and low expression in different genes. This situation also exists in different *Rafs* genes in rice. Maize *Rafs* (*GRMZM2G340656*) was highly expressed in 5DAP_cob, 5DAP_ovule, and R1_husk, with low expression in V2_stalk, V5_Tassel, V8_V9_ear, and V4_Tassel. *GRMZM2G150906* was highly expressed in 24DAP_root, VE_leaf, and V16_cob, with the lowest expression in V5_leaf. *GRMZM2G127147* exhibited relatively even expression in several tissues except for a slightly high expression in Anthers_TV_. The relatively high expression of these four *Rafs* genes in VE_leaf could be a consequence of the hormone-specific motif (i.e., CGTCA-motif) cooccurred in maize. For the *Rafs* gene in rice, the overall gene expression was similar except that *LOC_Os08g38710* was highly expressed. *LOC_Os08g38710* exhibited relatively even expression in several tissues except for the highest expression in young leaves. The expression levels of *LOC_Os07g10840* and *LOC_Os01g07530* were similar in some tissues; they were both expressed lowest in SAM. In contrast, *LOC_O07g10840* lacks some relevant detectable motifs, suggesting the presence of novel cis-elements in its promoter region and divergent expression regulation after gene duplication.

To further explore the potential functions of *Rafs* genes under drought and salt stress, we extracted RNA from the switchgrass variety alamo after stress treatment, reverse transcribed the plant RNA into cDNA, and performed real-time fluorescence quantitative analysis and visualization of the data. We subsequently analyzed the expression patterns of 16 switchgrass *Rafs* genes (and included all of their alternative transcripts) (Figure 9b). The results showed that the expression of all switchgrass *Rafs*, including both primary and alternative transcripts, was up-regulated under drought stress. The most up-regulated gene was *Pavir.2NG127100*, indicating that this gene may play a more important role in drought stress. Except for *Pavir.3NG191300.2*, *Pavir.7NG251200*, *Pavir.9NG061500.1,* and *Pavir.9 NG061500.2*, the expression of other *Rafs* genes and alternative transcripts was also up-regulated under salt stress. Together, these results indicated that most of the *Rafs* genes were up-regulated in switchgrass under drought and salt stress, suggesting that they might play an important role in improving the ability of switchgrass to resist drought and salt stress. Interestingly, the transcripts of *Pavir.9NG061500* and *Pavir.3NG191300* expressed differently under drought and salt stress, suggesting that the transcripts of the two genes may have different function.

**Figure 10:**
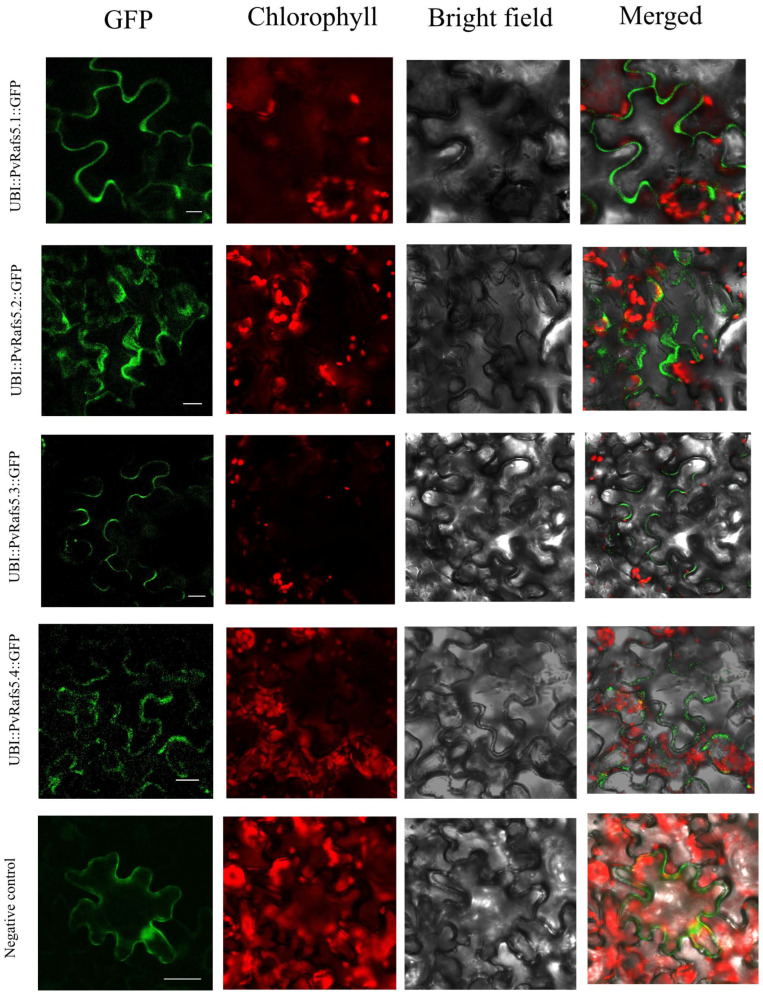
Subcellular localization of *Rafs* genes. Subcellular localization of *PvRafs5* and empty vector in tobacco leaves after 2 days of infiltration and 24 h of dark treatment showed that the green fluorescence of GFP was indicated. Scale bars = 25 µm.

Next, we use the sequence of *Pavir.9NG061500* gene to construct vectors, aim at determining the effect of AS on *Rafs* subcellular localization. Transient expression of *UBI::PvRafs5.1::GFP*, *UBI::PvRafs5.2::GFP*, *UBI::PvRafs5.3::GFP*, and *UBI::PvRafs5.4::GFP* plasmids showed that GFP fluorescence was mostly expressed in the cytoplasm or cell membrane (Figure 10), which may be consistent with its functional characteristics in regulating raffinose synthesis.

## 3. Discussion

Raffinose family oligosaccharides, especially raffinose, play important roles in plant growth and development. These include seed germination, carbon storage, and most importantly, plant stress tolerance [23]. *Rafs* is a key gene controlling the last step of raffinose synthesis and has been reported in many plants such as Arabidopsis [21], pea [24] and rice [26]. Moreover, genome-wide functional analysis of *Rafs* has also been reported in maize [32] and *Camellia sinensis* (white tea) [33]. Under stressed conditions, such as salinity, drought, heat, and cold, some grass species can accumulate raffinose as a defense [8,34,35,36]. However, there are few studies on the function of *Rafs* in grasses. Therefore, in this study, we conducted a comprehensive and systematic analysis of the *Rafs* gene family of grass species and explored the expression level during salinity and drought stress.

### 3.1. Evolutionary Analysis of the Rafs Gene Family

In this report, we successfully characterized 73 *Rafs* genes from 10 various grass species. Based on the evolutionary tree, we divided the selected *Rafs* into six clusters, and 73 *Rafs* genes were irregularly distributed in six clusters (Figure 1). Similarly, the *Rafs* evolutionary trees constructed in maize [32] and white tea [33] were also divided into six clusters. We observed that 16% of the *Rafs* genes had 2 exon structures, 27% had 6–7 exon structures, and 39% had more than 10 exon structures. In our study, the sequences containing Raffinose_syn domains were selected for phylogenetic construction. Multiple *Rafs* genes were found in almost all of these grasses, indicating the functional redundancy of *Rafs* genes. Whole genome duplication (WGD) also plays an important role in the *Rafs* gene family, which leads to the potentially diverse functions of *Rafs*. However, the mechanisms underlying the function of each *Rafs* gene still need to be further investigated. It has been suggested that overexpression of *GRMZM2G150906* can improve plant drought tolerance by synthesizing more raffinose [27]. *Pavir.5NG070900*, *Pavir.5NG075200*, *Pavir.5KG072800*, *Pahal.5G489000*, *Sevir.5G116500*, *Seita.5G119800*, *Sobic.003G052300*, *Oropetium_20150105_07232A*, *LOC_Os01g07530*, *Bradi2g04310*, *Brast01G370300*, and *GRMZM2G150906* from maize are clustered in Group 6 (Figure 1), and they also have similar gene structures (Figure 4), indicating that these proteins may have similar functions. Therefore, we speculate that these species may also be involved in raffinose accumulation under drought stress.

### 3.2. AS of Rafs in Grass

In plants, it is now well known that AS play important role in regulating the production of variant transcripts and increasing protein isoforms [37]. In this study, we found that AS significantly increased the transcriptome diversity of the grasses, with approximately 26% of genes undergoing AS. Furthermore, exon skipping was the most common AS event (89%). Second, alternative 5′ sites and alternative 3′ sites accounted for 52% and 15%, respectively. Intron retention events accounted for the proportion 47%. AS profile analysis showed that *Pavir.9NG061500*, *Pavir.6KG338200*, *Pavir.6KG336200*, and *Pavir.3NG191300*—their proteins expressed by isoforms designated major transcripts—showed similar patterns of protein motif distribution (Figure 7). However, their other transcripts have deletions of protein motifs through exon skipping and AS of the transcription start region, which may lead to alterations in *Rafs* protein function. In addition to increasing protein isoforms with altered sequences and functions, As is able to control mRNA levels by coupling to nonsense-mediated mRNA decay (NMD), thus decrease the frameshifted or truncated proteins. Splicing transcripts resulted from AS contain premature termination codons (PTCs) or long 3′-UTRs, which potentially make them for degradation by NMD [38,39]. Among the 19 *Rafs* genes with alternatively spliced mRNA transcripts, *Bradi1g54210.2*, *Pavir.6KG338220.2*, *Pavir.6KG336200.2*, *Pavir.3NG191300.2*, *GRMZM2G037265.2*, *GRMZM2G037265.3*, and *GRMZM2G037265.6* are potentially subjected to NMD. However, more experimental evidence needed to confirm whether these transcripts are turned over by NMD. Analysis of AS of *Rafs* gene in grass will make the study of raffinose synthesis much more extended through overexpression or knockout of specific isoforms of interest.

### 3.3. Different Expression Patterns and Cis-Element Analysis to Predict the Potential Function of Rafs

The expression patterns of *Rafs* were different in different tissues. In rice, *LOC_Os08g38710* was expressed relatively uniformly in other tissues, except in young leaves, where its expression was highest. This indicates that this gene may play a role in plant leaf growth. *LOC_Os07g10840*, *LOC_Os01g07530*, and *LOC_Os03g59430* were expressed at high levels in seeds but at low levels in all other tissues (Figure 9a), suggesting that these three genes may play a role in seed germination. The different expression patterns illustrate that *Rafs* genes may have different functions in various tissues and developmental processes in plants.

Binding of transcription factors and cis-elements upstream of the promoter region can regulate gene transcription and ultimately lead to gene expression. We found that the various cis-elements in the promoter regions of these *Rafs* genes were mostly associated with plant hormone response elements and abiotic stress response elements (Figure 5). Studies have shown that gibberellin (GA) expression levels are related to plant tolerance to drought stress [40]. *Rafs* genes in switchgrass, such as *Pavir.7NG251200* and *Pavir.9NG061500*, have promoter cis-elements P-box and GARE-motif associated with GA responsiveness. *Pavir.3NG191300*, *Pavir.4KG086000*, *Pavir.5NG070900*, *Pavir.5NG075200* and *Pavir.7NG230500* have MBS cis-elements, and this cis-element is associated with drought inducibility. Their expression patterns also indicated that the expression of *Rafs* genes was up-regulated under drought stress (Figure 9b). Therefore, we predicted that these genes might have the ability to promote drought tolerance in switchgrass. Methyl jasmonate (MeJA) can alleviate the harmful effects of salt stress on rice and *Triticum aestivum* (wheat) [41,42]. The CGTCA motif is a cis-element involved in the reaction with MeJA. The *Rafs* gene expression patterns of switchgrass, *Pavir.2NG127100*, *Pavir.2KG440200*, and *Pavir.4KG086000* were up-regulated under salt stress and were the three genes with the highest number of CGTCA-motif cis-elements. We speculate that MeJA may be involved in transcription factor-cis-element binding to regulate the expression of *Rafs*. Therefore, we hypothesized that these *Rafs* genes have salt tolerance functions.

## 4. Materials and Methods

### 4.1. Rafs Sequence Collection and Identification from 10 Grass Species

The present study employed the protein sequence of Pavir.6KG336200 of *Rafs* (EC 2.4.1.82) as a reference to conduct BLAST [43] searches in the Phytozome v12.0 database (https://phytozome.jgi.doe.gov/pz/portal.html) (accessed on 8 January 2021) [44] to retrieve similar sequence collections (e-value cutoff = 1 × 10^−10^) from herbaceous grass genomes. To confirm the identity of *Rafs* proteins, SMART (http://smart.embl-heidelberg.de) (accessed on 12 January 2021) [45] was utilized, which detects the Raffinose_syn domain. The analysis resulted in the identification of 73 *Rafs* sequences from 10 grass species along with their corresponding transcript sequences and coding sequences for further analysis. Prediction of subcellular localization of the *Rafs* proteins was carried out using CELLO v.2.5 (http://cello.life.nctu.edu.tw) (accessed on 15 January 2021) [46]. Furthermore, the molecular properties of the 73 *Rafs* sequences, including their molecular weight, theoretical isoelectric point, instability coefficient, and hydrophilicity, were predicted using ExPASY (http://www.expasy.org/) (accessed on 30 January 2021) [47]. The secondary structure of *Rafs* was also predicted online using SOPMA (https://npsa-prabi.ibcp.fr/cgi-bin/npsa_automat.pl?page=npsa_sopma.html) (accessed on 30 January 2021) [48].

### 4.2. Phylogenetic Tree Construction of Rafs Gene Family in Grass Species

The present study employed the amino acid sequences of the aforementioned 73 *Rafs* genes for phylogenetic analysis. For genes with different transcript isoforms, the primary isoform was used. Multiple sequence alignments of all selected *Rafs* sequences were conducted using Muscle v3.8.31 [49]. Bayesian methods were utilized for constructing a rooted phylogenetic tree of the *Rafs* proteins, employing MrBayes 3.2 [50]. The phylogenetic trees were subsequently edited and visualized using FigTree v1.4.3 [51].

### 4.3. Gene Structure, Protein Domains, Protein Motifs, Conserved Motifs and Cis-Element Analysis

The genomic sequences and CDSs of 10 species were downloaded from the Phytozome v12.0 database [44]. The gene structures were visualized using the online Gene Structure Display Service (http://gsds.gao-lab.org/) (accessed on 8 December 2022) [52], and protein domains were predicted using SMART (http://smart.embl-heidelberg.de) (accessed on 8 February 2021) [45]. The CDS and protein sequences of all genes were subjected to conserved motif analysis using the MEME website (https://meme-suite.org/meme/) (accessed on 22 February 2021) [53], which considered the 12 most conserved motifs predicted for each sequence. The resulting motifs were visualized using TBtools [54,55]. The 1500 bp upstream sequence of the *Rafs* transcription initiation site was identified using TBtools. Putative cis-regulatory elements were predicted using the online program PlantCARE (http://bioinformatics.psb.ugent.be/webtools/plantcare/html/) (accessed on 2 March 2021) [56].

### 4.4. AS Profile and Conserved Splice Sites

All available alternative transcripts of *Rafs* genes were extracted from the Phytozome v12.0 database [44]. The alternative transcript structures were presented using the online Gene Structure Display Service (http://gsds.gao-lab.org/) (accessed on 12 December 2022) [52]. Sequences adjacent to the existing splicing sites, i.e., 20 bp on each side, were manually analyzed using BLAST. The consensus sequences of conserved splice sites were analyzed using Weblogo v3.0 software (https://weblogo.berkeley.edu/logo.cgi) (accessed on 27 March 2021).

### 4.5. Amino Acid Conservation Estimation and Protein Interaction Network Analysis

The AlphaFold structure of the rice *Rafs* protein (AF-Q5VQG4-F1) was downloaded from the AlphaFold Protein Structure database (https://alphafold.ebi.ac.uk/) (accessed on 25 June 2022) as a model. Amino acid conservation scores were calculated utilizing the ML method based on the ConSurf web server (https://consurf.tau.ac.il/) (accessed on 13 November 2022) [57]. Multiple sequence alignment and structural data were provided as input. Figures were drawn using PyMOL software v2.5.0 [58].

The peptide sequences from rice (*LOC_Os01g07530*) and purple false brome (*BRADI1G48050.1*) were used to generate the protein interaction network using the STRING program (https://cn.string-db.org/) (accessed on 23 October 2022). The most important parameters of this program were as follows: meaning of network edges, evidence; active interaction sources, experiments, and databases; minimum required interaction score, 0.400; and max number of interactors to show, no more than 10 interactions. Finally, predicted functional partners of *Rafs* proteins were presented in the form of an interaction network drawn by Cytoscape 3.9.1 software.

### 4.6. Expression Analysis of Rafs Genes in Representing Grass Species and Rafs Protein Subcellular Localization Assay

In this study, expression data for plant *Rafs* family members in maize and rice were retrieved from the Bio-Analytic Resource for Plant Biology (http://bar.utoronto.ca) (accessed on 27 April 2021). The logarithmic transformed values of the expression data were utilized to generate representative heatmaps for visualizing the expression levels of selected plant *Rafs* genes using TBtools [54,55].

Switchgrass Alamo seeds were sterilized by immersion in NaClO solution and rinsed with distilled water, and the treated seeds were transferred to Petri dishes containing moist filter paper and left in the dark until germination, during which time the seeds were kept moist. The germinated seeds were transferred to a 2:1 mixture of nutrient soil and vermiculite. One month later, they were randomly divided into three groups for the adversity stress treatment. Under drought stress, seedlings were subjected to drought for 10 days. Seedlings under salt stress were treated with 300 mmol/L NaCl solution for 24 h. Untreated seedlings were used as controls. All samples were immediately frozen in liquid nitrogen and stored at −80 °C. According to the manufacturer’s instructions, total RNA was extracted from all samples using the E.Z.N.A.^®^ Plant RNA Kit (Omega Bio-Tek, Guangzhou, China). For qRT-PCR analysis, first-strand cDNAs were synthesized from DNaseI-treated total RNA using the RT mix with DNase (All-in-One) (US EVERBRIGHT, Tianjin, China) according to the manufacturer’s instructions. qRT-PCR was performed on the applied biosystems PCR system using the 2 × HQ SYBR qPCR Mix (Low ROX) (ZOMANBIO, Beijing, China). The relative expression levels were calculated by using the 2^−∆∆Ct^ method. The qRT-PCR assays were performed with three biological and technical replicates. Finally, the data were generated into heatmaps to display the expression levels of switchgrass *Rafs* genes using TBtools [54,55]. The gene-specific primers are listed in Table S4.

The four CDSs of *PvRafs5* (*Pavir9NG061500*) were cloned into the pEGOEPubi-H-GFP vector using double enzyme digestion. Then, the plasmid (*UBI::PvRafs5-GFP*) was transferred into Agrobacterium strain GV3101. This strain was injected into 1-month-old tobacco leaves. Finally, GFP fluorescence was detected by confocal laser scanning microscopy (Zeiss7 DUO NLO). The gene-specific primers are listed in Table S5.

## 5. Conclusions

In this study, we identified 73 *Rafs* genes from 10 grass species and provided a comprehensive analysis of the phylogenetic relationships between plant *Rafs* members as well as the transcriptional and posttranscriptional regulation of representative *Rafs* genes. Given the important roles of *Rafs* as essential genes that regulate resistance in plants, it will be worthwhile to carry out functional studies on *Rafs* genes in planta.

## Data Availability

Not applicable.

## Supplementary Materials

The supporting information can be downloaded at: https://www.mdpi.com/article/10.3390/ijms241311120/s1.
