# Supplementary material for: Identification and Alternative Splicing Profile of the Raffinose synthase Gene in Grass Species
Source: Int J Mol Sci. 2023 Jul 5;24(13):11120. doi: 10.3390/ijms241311120 (PMC10342692; doi:10.3390/ijms241311120)
Supplement: Supplementary file 1 [file ijms-24-11120-s001.zip › ijms-2442534-supplementary.pdf]

Table S1. *Rafs* genes identified from 10 plant species.

**Plant species**	**Common name**	**No. of sequences identified**	**Sequence ID**
Brachypodium distachyon	Purple false brome	5	Bradi3g39220<br>Bradi1g54210<br>Bradi1g48050<br>Bradi5g13570<br>Bradi2g04310
Brachypodium stacei	*	5	Brast03G265700<br>Brast06G177200<br>Brast07G049600<br>Brast09G120600<br>Brast01G370300
Oropetium thomaeum	*	6	Oropetium_20150105_20308A<br>Oropetium_20150105_26753A<br>Oropetium_20150105_17382A<br>Oropetium_20150105_17380A<br>Oropetium_20150105_04775A<br>Oropetium_20150105_07232A
Oryza sativa	Rice	6	LOC_Os08g38710<br>LOC_Os07g10840<br>LOC_Os06g07600<br>LOC_Os04g40520<br>LOC_Os03g59430<br>LOC_Os01g07530
Panicum hallii	Hall's panicgrass	7	Pahal.6G249100<br>Pahal.9G040600<br>Pahal.2G094000<br>Pahal.4G310000<br>Pahal.4G309700<br>Pahal.7G192100<br>Pahal.5G489000
Panicum virgatum	Switchgrass	16	Pavir.6KG336200<br>Pavir.6KG338200<br>Pavir.3NG191300<br>Pavir.7NG251200<br>Pavir.9NG061500<br>Pavir.2NG127100<br>Pavir.4KG067600<br>Pavir.4KG086000<br>Pavir.2KG440200

			Pavir.7KG206700<br>Pavir.7NG230500<br>Pavir.4KG068000<br>Pavir.5KG072800<br>Pavir.5NG075200<br>Pavir.5NG070900<br>Pavir.J666000
Setaria italica	Foxtail millet	7	Seita.6G195300<br>Seita.9G043300<br>Seita.2G081000<br>Seita.4G057000<br>Seita.4G056900<br>Seita.7G139800<br>Seita.5G119800
Setaria viridis	Green foxtail	7	Sevir.6G202600<br>Sevir.9G042600<br>Sevir.2G084500<br>Sevir.4G054800<br>Sevir.4G054700<br>Sevir.7G148900<br>Sevir.5G116500
Sorghum bicolor	Sorghum	7	Sobic.007G219900<br>Sobic.001G044800<br>Sobic.002G075800<br>Sobic.010G057300<br>Sobic.010G057400<br>Sobic.006G122400<br>Sobic.003G052300
Zea mays Ensembl	Maize	7	GRMZM2G037265<br>GRMZM2G050177<br>GRMZM2G340656<br>GRMZM2G077181<br>GRMZM2G127147<br>GRMZM2G150906<br>GRMZM2G047292

Table S2. Predicted subcellular localization of plant Rafs proteins.

Species	Common name	Sequence ID	Start(bp)	End(bp)	Exon number	Genome length(bp)	Location	Protein length	Subcellular locations
Brachypodium distachyon	Purple false brome	Bradi3g39220	41325620	41330075	12	4456	Bd3	764	Chloroplast
		Bradi1g54210	52770572	52774678	6	4107	Bd1	760	Chloroplast
		Bradi1g48050	46832049	46835295	4	3247	Bd1	843	Chloroplast
		Bradi5g13570	17234227	17238371	14	4145	Bd5	817	Chloroplast
		Bradi2g04310	3048386	3051526	2	3141	Bd2	782	Chloroplast
Brachypodium stacei	*	Brast03G265700	22730251	22734699	13	4449	Chr03	759	Chloroplast
		Brast06G177200	16256632	16260522	6	3891	Chr06	763	Chloroplast
		Brast07G049600	2848079	2851208	4	3130	Chr07	838	Chloroplast
		Brast09G120600	11340548	11344725	14	4178	Chr09	826	Chloroplast
		Brast01G370300	27535251	27538468	2	3218	Chr01	793	Chloroplast
Oryza sativa	Japanese Rice	LOC_Os08g38710	24475146	24479529	13	4384	Chr8	753	Chloroplast
		LOC_Os07g10840	5890558	5894135	7	3578	Chr7	763	Chloroplast
		LOC_Os06g07600	3675572	3679002	6	3431	Chr6	841	Chloroplast
		LOC_Os04g40520	24068037	24072124	14	4088	Chr4	738	Cytoplasmic
		LOC_Os03g59430	33829500	33834198	7	4699	Chr3	505	Cytoplasmic
		LOC_Os01g07530	3598418	3602211	2	3794	Chr1	840	Chloroplast
Oropetium thomaeum	*	Oropetium_20150105_20308A	394209	398399	12	4191	Oropetium_genomic_20141112_068	796	Chloroplast
		Oropetium_20150105_26753A	21626	25880	9	4255	Oropetium_genomic_20141112_162	780	Chloroplast
		Oropetium_20150105_17382A	1599430	1602703	6	3274	Oropetium_genomic_20141112_046	763	Chloroplast
		Oropetium_20150105_17380A	1593581	1596610	6	3030	Oropetium_genomic_20141112_046	752	Chloroplast

		Oropetium_20150105_04775A	439549	442205	3	2657	Oropetium_genomic_20141112_003	759	Cytoplasmic
		Oropetium_20150105_07232A	2334833	2337593	2	2761	Oropetium_genomic_20141112_014	784	Chloroplast
Panicum hallii	Hall's panicgrass	Pahal.6G249100	41223928	41228643	13	4716	Chr06	753	Cytoplasmic
		Pahal.9G040600	2250671	2255416	10	4746	Chr09	766	Cytoplasmic
		Pahal.2G094000	7086625	7090709	5	4085	Chr02	770	Cytoplasmic
		Pahal.4G310000	49525471	49529836	7	4366	Chr04	863	Chloroplast
		Pahal.4G309700	49506203	49509327	6	3125	Chr04	764	Chloroplast
		Pahal.7G192100	37837014	37841211	14	4198	Chr07	806	Chloroplast
		Pahal.5G489000	55995024	55998693	2	3670	Chr05	786	Chloroplast
Panicum virgatum	Switchgrass	Pavir.6KG336200	64586278	64590416	13	4139	Chr06K	752	Cytoplasmic
		Pavir.6KG338200	64734106	64738224	13	4119	Chr06K	753	Cytoplasmic
		Pavir.3NG191300	35551910	35556245	13	4336	Chr03N	753	Cytoplasmic
		Pavir.7NG251200	47574333	47579250	10	4918	Chr07N	760	Cytoplasmic
		Pavir.9NG061500	3912768	3917385	10	4618	Chr09N	761	Cytoplasmic
		Pavir.2NG127100	19121861	19125008	4	3148	Chr02N	757	Cytoplasmic
		Pavir.4KG067600	6701727	6705531	7	3805	Chr04K	871	Chloroplast
		Pavir.4KG086000	8194685	8197891	6	3207	Chr04K	767	Chloroplast
		Pavir.2KG440200	78497128	78500277	3	3150	Chr02K	595	Periplasmic
		Pavir.7KG206700	51526138	51530319	14	4182	Chr07K	801	Chloroplast
		Pavir.7NG230500	49405119	49409124	14	4006	Chr07N	767	Cytoplasmic
		Pavir.4KG068000	6732371	6736022	5	3652	Chr04K	643	Cytoplasmic
		Pavir.5KG072800	9143696	9147501	2	3806	Chr05K	785	Chloroplast
		Pavir.5NG075200	8630083	8633983	2	3901	Chr05N	784	Chloroplast
		Pavir.5NG070900	8364401	8368341	2	3941	Chr05N	784	Chloroplast

		Pavir.J666000	5960	7633	5	1674	scaffold_6879	410	Cytoplasmic
Setaria italica	Foxtail millet	Seita.6G195300	31734112	31738431	13	4320	scaffold_6	753	Cytoplasmic
		Seita.9G043300	2429736	2434322	10	4587	scaffold_9	766	Cytoplasmic
		Seita.2G081000	7016721	7019764	3	3044	scaffold_2	779	Cytoplasmic
		Seita.4G057000	4220412	4224375	7	3964	scaffold_4	862	Chloroplast
		Seita.4G056900	4212770	4216250	6	3481	scaffold_4	765	Chloroplast
		Seita.7G139800	23049676	23054038	14	4363	scaffold_7	883	Chloroplast
		Seita.5G119800	10053068	10056645	2	3578	scaffold_5	782	Chloroplast
Setaria viridis	Green foxtail	Sevir.6G202600	32039498	32043922	13	4425	Chr_06	753	Cytoplasmic
		Sevir.9G042600	2448938	2453684	10	4747	Chr_09	766	Cytoplasmic
		Sevir.2G084500	7280326	7283520	3	3195	Chr_02	779	Cytoplasmic
		Sevir.4G054800	4248784	4252921	7	4138	Chr_04	862	Chloroplast
		Sevir.4G054700	4237703	4240951	6	3249	Chr_04	764	Chloroplast
		Sevir.7G148900	22159303	22163694	14	4392	Chr_07	882	Chloroplast
		Sevir.5G116500	9479721	9483530	2	3810	Chr_05	782	Chloroplast
Sorghum bicolor	Sorghum	Sobic.007G219900	64802754	64807148	13	4395	Chr07	754	Cytoplasmic
		Sobic.001G044800	3325484	3330705	10	5222	Chr01	773	Cytoplasmic
		Sobic.002G075800	7874437	7878146	6	3710	Chr02	764	Chloroplast
		Sobic.010G057300	4474788	4478602	7	3815	Chr10	857	Chloroplast
		Sobic.010G057400	4487128	4496678	7	9551	Chr10	738	Chloroplast
		Sobic.006G122400	48852708	48856765	14	4058	Chr06	810	Chloroplast
		Sobic.003G052300	4721513	4725477	2	3965	Chr03	792	Chloroplast
Zea mays Ensembl	Maize	GRMZM2G037265	183584834	183588867	13	4034	chr01	747	Chloroplast
		GRMZM2G050177	183697224	183704114	16	6891	chr01	922	Chloroplast

		GRMZM2G340656	19063721	19083446	7	19726	chr07	756	Cytoplasmic
		GRMZM2G077181	291025126	291029456	10	4331	chr01	745	Chloroplast
		GRMZM2G127147	122910609	122914067	7	3459	chr06	843	Chloroplast
		GRMZM2G150906	12571385	12575346	2	3962	chr03	790	Chloroplast
		GRMZM2G047292	139818315	139820635	8	2321	chr02	491	Chloroplast

Table S3. Physicochemical characteristics of plant Rafs proteins.

Species	Sequence ID	Molecular weight (kDa)	Theoretical pI	Negatively/Positively charged residues (%)	Instability index	Grand average of hydropathicity (GRAVY)	Alpha helix (%)	Extended strand (%)	Beta turn (%)	Random coil (%)
Brachypodium distachyon	Bradi3g39220	82.44	5.85	12.04/9.95	35.39	−0.148	27.49	22.25	7.72	42.54
	Bradi1g54210	82.77	5.69	11.58/9.34	81.33	−0.135	28.42	22.76	7.76	41.05
	Bradi1g48050	89.36	6.51	10.79/10.08	83.46	−0.041	27.88	22.18	7.00	42.94
	Bradi5g13570	88.69	5.7	11.02/9.18	85.30	−0.091	27.17	21.66	7.47	43.70
	Bradi2g04310	84.26	5.7	11.51/8.82	85.77	−0.008	27.11	22.12	5.88	44.88
Brachypodium stacei	Brast03G265700	81.83	6.18	11.46/10.01	80.82	−0.155	26.61	22.27	6.59	44.53
	Brast06G177200	82.73	5.56	11.80/9.04	81.66	−0.127	27.13	21.89	7.86	43.12
	Brast07G049600	88.67	6.79	10.62/10.26	82.26	−0.06	29.24	20.05	7.64	43.08
	Brast09G120600	89.89	5.66	10.90/8.84	83.29	−0.127	28.57	20.22	6.54	44.67
	Brast01G370300	84.77	5.85	10.84/8.83	84.14	0.001	27.87	21.94	7.19	43.00
Oryza sativa	LOC_Os08g38710	81.54	5.9	11.02/8.90	83.12	−0.105	27.49	22.31	7.30	42.90
	LOC_Os07g10840	82.01	5.45	12.06/8.91	81.07	−0.082	29.49	21.10	7.47	41.94
	LOC_Os06g07600	89.67	6.78	10.11/9.75	82.95	−0.036	26.99	20.69	6.66	45.66
	LOC_Os04g40520	81.19	5.30	12.20/8.94	84.43	−0.111	30.35	22.09	7.18	40.38
	LOC_Os03g59430	54.91	5.55	11.09/7.92	82.12	−0.032	36.04	16.83	5.54	41.58
	LOC_Os01g07530	91.92	6.26	11.55/10.48	80.45	−0.13	27.26	22.26	6.31	44.17
Oropetium thomaeum	Oropetium_20150105_20308A	87.26	6.24	10.93/9.67	85.20	−0.134	27.51	22.36	5.90	44.22
	Oropetium_20150105_26753A	84.37	*	12.18/9.74	79.23	−0.2	29.23	21.41	6.54	42.82
	Oropetium_20150105_17382A	81.59	5.89	11.27/9.44	84.64	−0.048	28.57	21.36	7.21	42.86

	Oropetium_20150105_17380A	79.97	6.04	10.37/9.04	85.49	0.043	27.79	23.01	7.98	41.22
	Oropetium_20150105_04775A	70.24	*	10.67/8.43	65.24	−0.157	29.12	22.40	7.51	40.97
	Oropetium_20150105_07232A	85.48	6.06	12.12/10.71	79.95	−0.12	27.04	22.96	6.38	43.62
Panicum hallii	Pahal.6G249100	82.31	6.15	11.02/9.56	82.03	−0.163	28.55	22.18	6.77	42.50
	Pahal.9G040600	83.55	5.89	11.75/9.53	80.22	−0.173	28.85	21.54	7.05	42.56
	Pahal.2G094000	82.38	5.58	12.08/8.96	80.35	−0.119	27.27	21.95	8.31	42.47
	Pahal.4G310000	91.63	6.73	10.08/9.73	81.07	−0.073	26.88	21.78	6.72	44.61
	Pahal.4G309700	81.18	5.59	11.65/9.16	82.53	−0.021	28.93	21.47	7.33	42.28
	Pahal.7G192100	89.1	6.22	11.17/9.93	82.67	−0.163	29.78	21.71	7.57	40.94
	Pahal.5G489000	85.31	5.87	11.70/9.92	81.26	−0.098	27.23	21.25	6.87	44.66
Panicum virgatum	Pavir.6KG336200	82.09	6.3	11.04/9.97	82.14	−0.168	27.39	22.07	7.18	43.35
	Pavir.6KG338200	82.13	6.23	11.02/9.83	82.03	−0.159	28.02	23.24	6.91	41.83
	Pavir.3NG191300	82.14	6.09	11.16/9.56	82.55	−0.161	26.96	22.44	7.44	43.16
	Pavir.7NG251200	83.03	5.84	11.97/9.74	81.63	−0.155	27.37	22.11	6.58	43.95
	Pavir.9NG061500	82.8	5.76	11.70/9.33	81.02	−0.161	27.33	22.08	7.36	43.23
	Pavir.2NG127100	81.12	5.86	11.36/9.38	81.45	−0.051	27.61	22.99	7.00	42.40
	Pavir.4KG067600	92.69	6.88	9.99/9.76	80.68	−0.098	25.03	21.13	6.77	47.07
	Pavir.4KG086000	81.43	5.62	11.47/9.26	84.11	−0.006	26.86	22.56	7.30	43.29
	Pavir.2KG440200	64	6.49	11.09/10.25	75.61	−0.204	34.45	17.98	5.21	42.35
	Pavir.7KG206700	88.47	6.31	11.36/10.36	83.56	−0.183	29.34	20.85	6.87	42.95
	Pavir.7NG230500	84.34	5.67	11.73/9.26	84.59	−0.156	27.77	21.64	7.04	43.55
	Pavir.4KG068000	69.67	9.68	10.58/13.22	73.95	−0.368	33.75	16.95	7.47	41.84
	Pavir.5KG072800	85.12	6.08	11.46/10.06	80.74	−0.089	28.54	22.17	6.11	43.18
	Pavir.5NG075200	85.05	6.12	11.48/10.20	84.20	−0.05	27.17	21.05	7.02	44.77

	Pavir.5NG070900	85.16	6.12	11.48/10.20	84.06	−0.056	27.55	21.17	7.14	44.13
	Pavir.J666000	44.06	6.21	10.49/8.78	88.71	−0.047	23.90	24.63	7.56	43.90
Setaria italica	Seita.6G195300	82.27	6.01	11.29/9.43	82.93	−0.15	27.22	22.05	7.57	43.16
	Seita.9G043300	83.38	5.63	14.36/10.97	81.92	−0.11	27.15	22.32	7.31	43.21
	Seita.2G081000	83.03	5.74	11.81/9.50	78.41	−0.134	29.40	21.57	9.50	39.54
	Seita.4G057000	91.78	6.63	10.32/9.86	82.87	−0.071	27.03	21.23	6.38	45.36
	Seita.4G056900	81.43	5.59	11.37/8.63	83.93	0.009	28.89	21.57	7.06	42.48
	Seita.7G139800	97.51	6.76	11.10/10.76	79.99	−0.254	25.93	19.93	7.81	46.32
	Seita.5G119800	85.12	5.85	11.89/9.97	80.68	−0.095	27.49	20.72	6.01	45.78
Setaria viridis	Sevir.6G202600	82.28	6.01	11.29/9.43	82.30	−0.163	27.49	22.58	7.44	42.50
	Sevir.9G042600	83.39	5.63	12.01/9.40	80.97	−0.157	27.42	21.54	7.05	43.99
	Sevir.2G084500	83.03	5.69	11.94/9.50	78.41	−0.134	29.27	20.92	8.86	40.95
	Sevir.4G054800	91.92	6.53	10.44/9.86	83.20	−0.074	27.15	21.35	6.61	44.90
	Sevir.4G054700	81.37	5.51	11.52/8.64	83.66	0.001	27.75	21.20	6.68	44.37
	Sevir.7G148900	97.36	6.66	11.11/10.66	80.52	−0.247	26.87	19.50	7.14	46.49
	Sevir.5G116500	85.13	5.85	11.89/9.97	80.55	−0.097	25.45	20.97	6.39	47.19
Sorghum bicolor	Sobic.007G219900	82.36	6.1	11.01/9.55	80.89	−0.187	28.12	23.08	7.43	41.38
	Sobic.001G044800	83.73	5.85	11.77/9.70	78.95	−0.18	28.46	21.09	6.73	43.73
	Sobic.002G075800	82.34	6.03	11.52/9.82	79.92	−0.135	25.92	22.51	7.46	44.11
	Sobic.010G057300	91.23	6.51	10.50/9.92	82.21	−0.071	26.84	20.89	6.65	45.62
	Sobic.010G057400	78.64	6.07	10.57/9.21	84.63	0.018	29.54	21.41	7.45	41.60
	Sobic.006G122400	89.3	5.86	11.60/9.75	86.00	−0.124	28.52	21.11	7.53	42.84
	Sobic.003G052300	85.98	5.8	11.87/9.85	80.90	−0.095	25.25	21.46	5.93	47.35
	GRMZM2G037265	81.17	6.59	10.44/9.77	82.45	−0.146	26.77	23.03	7.23	42.97

Zea mays Ensembl	GRMZM2G050177	100.38	6.65	10.74/10.20	80.65	−0.196	26.14	22.89	8.79	42.19
	GRMZM2G340656	81.93	5.95	11.51/9.52	80.99	−0.13	29.50	22.22	7.80	40.48
	GRMZM2G077181	80.85	5.93	11.41/9.40	80.36	−0.125	27.79	21.88	6.98	43.36
	GRMZM2G127147	90.61	6.12	11.15/9.96	84.26	−0.079	28.47	21.23	7.83	42.47
	GRMZM2G150906	85.59	5.86	11.77/9.75	81.95	−0.082	26.58	20.63	6.33	46.46
	GRMZM2G047292	54.09	8.23	10.79/11.41	86.21	−0.161	36.05	15.07	6.72	42.16

Table S4. qRT-PCR primer information of switchgrass *Rafs* gene

Gene ID	Gene name	Primer sequence (5′- 3′)	Primer fragment (bp)
Pavir.6KG336200.1	Rafs1.1-F	ACCTTGTCCATGATGAGCAGCC	22
	Rafs1.1-R	CTCTCCTCCGACGTGCGAATA	21
Pavir.6KG336200.2	Rafs1.2-F	ACCTTGTCCATGATGAGCAGCC	22
	Rafs1.2-R	GCAGAAATGATGATTTACCTCCGAC	25
Pavir.6KG338200.1	Rafs2.1-F	ATCTTGCATCTGCTGGGATCGA	22
	Rafs2.1-R	TCTCCTCCGACGTGCGAATACA	22
Pavir.6KG338200.2	Rafs2.2-F	AAGAACCTTGTCCATGATGAGCAG	24
	Rafs2.2-R	TATGGACAGCAGATTGCTTGTCAT	24
Pavir.6KG338200.3	Rafs2.3-F	GGTGTTGTTGGAATGGAGCAATA	23
	Rafs2.3-R	AGCACCTAGTGTCTCAAGGATGTTT	25
Pavir.3NG191300.1	Rafs3.1-F	CGGGTAGGGAAGAAGAACCTTGTT	24
	Rafs3.1-R	CACCTCTCCTCCTACGTGCGAAT	23
Pavir.3NG191300.2	Rafs3.2-F	CGGGTAGGGAAGAAGAACCTTGTT	24
	Rafs3.2-R	TTAATGACAAGCAATCTGCTGTCCA	25
Pavir.7NG251200	Rafs4-F	AGTTCACCTACGACGCCGACA	21
	Rafs4-R	TCCACCAAAGAATGAGGCGAG	21
Pavir.9NG061500.1	Rafs5.1-F	GCAGCAGATCGGCAGCGAGAACAA	24
	Rafs5.1-R	TCTGCTGCTGCTCTGCCTCCTCCTT	25
Pavir.9NG061500.2	Rafs5.2-F	GCAGCAGATCGGCAGCGAGAACAA	24
	Rafs5.2-R	CGCGCTTCGCCTCCTCCTTCTTGTT	25
Pavir.9NG061500.3	Rafs5.3-F	TGTAGCAGAAATGCTCGGTTCTGA	24
	Rafs5.3-R	TTGTCCCTGTGGTGGAACGTC	21
Pavir.9NG061500.4	Rafs5.4-F	CACTCACGTCACGTTCTGAAACTTT	25
	Rafs5.4-R	CATTGGCCTCCTCCTTGTTCTC	22
Pavir.2NG127100	Rafs6-F	AGATCACGGTGTAGGCCGAGA	21
	Rafs6-R	AGGCACATCACAATCACCCTCA	22
Pavir.4KG067600	Rafs7-F	CCCAAGGAGAGGGTTCACAAGAT	23
	Rafs7-R	TCCAGTCCCTACCATCAACATGC	23
Pavir.4KG086000	Rafs8-F	TCGGCTGAGATCGAGTTCTCCTA	23
	Rafs8-R	ACCGCACCGCAAGTCTACAACT	22
Pavir.2KG440200	Rafs9-F	ATTCTCAGCCGTCGCCATTG	20
	Rafs9-R	ACACCACAGTGGCATAGCAAAGC	23
Pavir.7KG206700	Rafs10-F	CAGTCATACAGAGGATGGCCTGC	23
	Rafs10-R	AATGGCAGGAAATCGCCTGTT	21
Pavir.7NG230500	Rafs11-F	GCCATTGTTCAGTTGGTTCTGATG	24
	Rafs11-R	ATCGTGACAGAATTGGCGCTG	21
Pavir.4KG068000	Rafs12-F	AGCATCAGCAATCCAGAACACCA	23
	Rafs12-R	AGGCATCAGTTCATATCGCCGA	22
Pavir.5KG072800	Rafs13-F	GTTCGCCTACAAGGACGGCAT	21
	Rafs13-R	ACGGACTGATAGGCGAATGCAC	22
Pavir.5NG075200	Rafs14-F	TTCGCCTACAAGGACGGCTTG	21

	Rafs14-R	AGCCTGATAGGCGAATGTGACGT	23
Pavir.5NG070900	Rafs15-F	GTTCGCCTACAAGGACGGCTT	21
	Rafs15-R	AGCCTGATAGGCGAATGTGACG	22
Pavir.J666000	Rafs16-F	CGGATTGGTGTGCATGGAAGT	21
	Rafs16-R	ATCTTGTGAACCCTCTCCTTGGG	23
	Actin-F	AGCAGCATGAAGATCAAGGTGGTT	24
	Actin-R	CGGACTCATCGTACTCAGCCTTG	23

Table S5. Primers used to construct the *UBI::PvRafs-GFP* vector.

Gene ID	Gene name	Primer sequence	Primer fragment (bp)
Pavir.9NG061500.1	PVRafs5.1-F	ATGACGGTGACGCCGAAGATCACG	24
	PvRafs5.1-R	CCATCTGTAGAACTCCTTCTCCGGCAC	27
Pavir.9NG061500.2	PvRafs5.2-F	ATGACGGTGACGCCGAAGATCACGG	25
	PvRafs5.2-R	GACCTGGATCTCCAGGTTCCATCTGTACAACTCC	34
Pavir.9NG061500.3	PvRafs5.3-F	ATGGACAGGGTTGTGGAGAGGCACCT	26
	PvRafs5.3-R	GACCTGGATCTCCAGGTTCCATCTGTACAACTCCT	35
Pavir.9NG061500.4	PvRafs5.4-F	ATGGCGGGGTACTGGGGCGG	20
	PvRafs5.4-R	GACCTGGATCTCCAGGTTCCATCTGTACAACTCCTGC	37
